# Molecular interactions of Bcl-2 and Bcl-xL with mortalin: identification and functional characterization

**DOI:** 10.1042/BSR20130034

**Published:** 2013-10-16

**Authors:** Nishant Saxena, Shashank P. Katiyar, Ye Liu, Abhinav Grover, Ran Gao, Durai Sundar, Sunil C. Kaul, Renu Wadhwa

**Affiliations:** *National Institute of Advanced Industrial Science and Technology (AIST), Central 4, 1-1-1 Higashi, Tsukuba Science City 305-8562, Japan; †Department of Biochemical Engineering and Biotechnology, Indian Institute of Technology (IIT) Delhi, Hauz Khas, New Delhi 110016, India

**Keywords:** Bcl-2, Bcl-xL, interaction, mortalin, p53 activation, senescence, Bad, Bcl-2/Bcl-xL-antagonist, causing cell death, BH, Bcl-2 homology, Bim, Bcl-2-interacting mediator of cell death, DMEM, Dulbecco’s modified Eagle’s medium, GFP, green fluorescent protein, Hsp 70, heat-shock protein 70, IC, immunocomplexes, MD, molecular dynamics, PBS-T, Triton X-100 in PBS, PT, permeability transition, ROS, reactive oxygen species

## Abstract

Bcl-2 family of proteins consists of both pro-apoptotic and anti-apoptotic members that control cellular apoptosis. They predominantly reside in the mitochondria and control the release of apoptotic factors from the mitochondria to the cytosol by regulating its membrane potential and opening the PT (permeability transition) pore. Here we report bioinformatics and biochemical evidence to demonstrate the interaction between Bcl-2 and Bcl-xL with a stress chaperone, mortalin. We demonstrate that such interaction results in the abrogation of mortalin-p53 interaction leading to nuclear translocation and transcriptional reactivation of p53 function that results in an induction of senescence in cancer cells.

## INTRODUCTION

The Bcl-2 family proteins, of which Bcl-2 was first identified as an oncoprotein contributing to B-cell lymphoma, have been established as regulators of apoptosis. They fall into two groups, one consisting of anti-apoptotic proteins such as Bcl-2, Bcl-xL, Bcl-w, Mcl-1, and the other consisting of pro-apoptotic proteins including Bcl-xS, Bax, Bak, Bid and Bad (Bcl-2/Bcl-xL-antagonist, causing cell death). They all have up to four conserved α-helical segments, called BH (Bcl-2 homology) domains (BH1–4). Whereas the first N-terminal BH4 domain is important for anti-apoptotic activity and is less conserved in pro-apoptotic members, BH3 domain has been shown to be crucial for cell-death activity of pro-apoptotic members. Functions of these proteins, such as control of mitochondrial membrane permeability, release of mitochondrial apoptogenic factors into the cytoplasm and regulation of apoptosis, depend on (i) their capacity to homo- or hetero-dimerize with BH3 domains and (ii) integration of the carboxy-terminal hydrophobic domain, or TM (transmembrane) domain, into specific cytoplasmic membranes [1,2].

Bax, Bcl-2 and Bcl-xL are abundantly present in the mitochondrial PT (permeability transition) pore/mitochondrial megachannel (a multiprotein complex formed at the contact site between the mitochondrial inner and outer membranes). The PT pore regulates matrix Ca^2+^, pH, membrane conductance and bioenergetics of mitochondria. Whereas the pro-apoptotic Bcl-2 family proteins [Bak, Bax and Bim (Bcl-2-interacting mediator of cell death)] are required for disruption of mitochondria structure and function, release of cytochrome *c* into the cytosol and cell death, the anti-apoptotic proteins (Bcl-2, Bcl-xL and Mcl-1) function to preserve mitochondrial integrity and prevent the loss of mitochondrial membrane potential and cell death by interfering with the action of Bax and Bak. They are overexpressed in many cancers in contrast with the pro-apoptotic proteins (Bad, Bak, Bax, Bid, Bim and Bcl-xS, transcribed as a small protein by alternative splicing from Bcl-X mRNA, also encoding Bcl-xL) that are either lost or under expressed [1]. Cancers with high level of Bcl-2 or Bcl-xL are also resistant to a wide spectrum of chemotherapeutic agents and radiation therapy. Based on these studies, they were proposed as attractive anti-cancer targets. The small-molecule inhibitors that block the anti-apoptotic function of Bcl-2 or Bcl-xL leading to recurrence of apoptosis in cancer cells have been proposed as potential new anti-cancer agents [3,4]. Pro-survival and cell cycle progression activities of Bcl-2 are regulated by its post-translational modifications and signal transduction by protein kinases, also called Bcl-2 kinases. It has been shown that phosphorylation of Bcl-2 at Ser^70^ (single site phosphorylation) is required for anti-apoptotic function of Bcl-2. However, multisite phosphorylation inhibits Bcl-2 suggesting the potential wide range of functional consequences of its post-translational modification [5].

Mortalin/mtHsp70/PBP74/Grp75 (mot-2) is a member of the Hsp 70 (heat-shock protein 70) family, predominantly present in mitochondria and involved in mitochondrial import, control of membrane permeability and ROS (reactive oxygen species) production [6–9]. Pro-proliferative function of mortalin (mot-2) has been demonstrated by several studies including its overexpression resulting in (i) lifespan extension of normal human fibroblasts [8,9], (ii) growth advantage and attenuation of differentiation of HL-60 promyelocytic leukaemia cells [10], (iii) malignant transformation of mouse and human immortal cells [11,12] and (iv) lifespan extension of worms [13]. On the other hand, suppression of mortalin caused growth arrest in human cancer cells and progeria-like phenotype in worms [14–16]. The pro-proliferative effects of mortalin in cancer cells have been assigned, at least in part, to its binding with the tumour suppressor protein, p53 that results in its retention in the cytoplasm and inhibition of its transcriptional activation and control of centrosome duplication function [17–19]. Several other studies have assigned an anti-apoptotic function to mortalin [20–23].

Besides its well-established nuclear localization, p53 is also located in mitochondria where it mediates transcription-independent tumour suppression by induction of mitochondrial permeabilization and apoptosis [24–25]. p53 has been shown to interact with Bcl-2, Bcl-xL and mortalin (mot-2). Whereas the interaction of Bcl-2 and Bcl-xL involves its DNA binding domain [26], the interaction with mortalin occurs at the cytoplasmic sequestration domain [18]. Apoptosis induced by DNA damage was shown to involve p53-Bcl-2 interactions that led to abrogation of Bcl-2 and Bax binding. p53-induced production of ROS and cellular senescence was also inhibited by Bcl-xL [27]. Mitochondrial p53 was shown to complex with Bcl-2 and Bcl-xL resulting in cytochrome *c* release and apoptosis. Of note, p53 mutants found in tumours are defective in their binding to Bcl-xL implying that inhibition of p53-mediated apoptosis may contribute to continued survival of tumour cells. The interaction of p53 and Bcl-xL is found to be blocked by binding of a 25-residue peptide derived from the BH3 region of the pro-apoptotic Bad protein [26]. Similarly, the carboxy-terminal peptide of p53 is able to abrogate mortalin-p53 interactions resulting in growth arrest in cancer cells [18].

In view of the above described interaction of Bcl-2, Bcl-xL and mortalin with p53 and their role as anti-apoptotic proteins, we suspected that these proteins might form a complex having functional consequences on either proliferative or apoptotic phenotype of cells. We undertook bioinformatics and biochemical studies and demonstrated that Bcl-2 and Bcl-xL interact with mortalin and activate p53 function leading to an induction of senescence in cancer cells.

## EXPERIMENTAL

### Plasmids, cell culture, transfections and antibodies

The cDNA encoding full length Bcl-xL protein was amplified from human bone marrow total RNA by RT–PCR (reverse transcription–PCR) using Bcl-xL specific primers (sense: 5′ ATG TCT CAG AGC AAC CGG GA 3′ and antisense: 5′ TCA TTT CCG ACT GAA GAG TGA 3′). The amplified cDNA was cloned into pcDNA3.1 plasmid (Invitrogen) resulting in expression of V5-tag at the carboxy-terminus of the protein. The expressed protein was thus detected by Western blotting with anti-V5 tag antibody. Plasmid expressing GFP (green fluorescent protein)-tagged Bcl-2 protein was obtained from Addgene. Protein expression was observed as either direct GFP fluorescence under the microscope or by Western blotting with anti-GFP antibody.

Cells (U2OS; osteosarcoma) were cultured in DMEM (Dulbecco's modified Eagle's medium) essential medium supplemented with 10% (v/v) FBS (fetal bovine serum) and incubated in a 5% CO_2_/95% air humidified incubator. Plasmid DNA transfections were performed using Fugene 6 (Roche Diagnostics). Typically, 3 μg of plasmid was used per well in a 6-well dish. Cell lysates were prepared in Nonidet P40 lysis buffer [10 mM Tris/HCl (pH 7.4), 150 mM NaCl, 5 mM EDTA, 1% Nonidet P40] supplemented with a protease inhibitor cocktail (Complete™ Mini; Roche Diagnostics) following 48 h of transfection. Stable cells lines were obtained by 2 weeks of selection for the transfected gene using 800 μg/ml Gly^418^ (Invitrogen) in the medium, and then maintained with 200 μg/ml Gly^418^ in DMEM.

The antibodies were purchased from the following sources: monoclonal anti-Bcl-xL (Cell Signaling), monoclonal anti-V5 (Invitrogen), anti-p21WAF1 (C-19) (Santa Cruz Biotech), anti p53 DO-1 (Santa Cruz), anti-p53 CM1 (Novocastra) and monoclonal anti-actin (Chemicon).

### Immunoprecipitation

Cell lysates (250 μg of protein) in 400 μl Nonidet P40 lysis buffer were incubated with the indicated polyclonal antibody overnight at 4°C. IC (immunocomplexes) were separated by incubation with 20 μl of Protein-A/G plus Agarose (Santa Cruz Biotech) for 1 h and centrifuged. IC was thoroughly washed with Nonidet P40 buffer and resolved on SDS/10%PAGE, electro-blotted onto a PVDF membrane (Millipore Corporation) using a semi-dry transfer system (Biorad). The proteins in IC were detected by Western blotting with the indicated monoclonal antibody and viewed using ECL (enhanced chemiluminescence, Amersham Pharmacia Biotech).

### Immunostaining

Cells grown to about 60% confluency, on glass coverslips placed in 12-well plastic dishes, were washed with cold PBS and fixed with a pre-chilled methanol:acetone (1:1, v/v) mixture for 5 min on ice. Fixed cells were washed with PBS, permeabilized with 0.2% PBS-T (Triton X-100 in PBS) for 10 min, and blocked with 2% BSA in PBS for 20 min. Cells were probed for Bcl-xL, mortalin and p53 using the indicated antibodies, and counterstained with Hoechst 33258 (Invitrogen). The antibody staining was visualized by incubation with secondary antibodies, donkey anti-goat IgG (Alexa Fluor® 488-conjugated) or donkey anti-rabbit IgG (Alexa Fluor® 594-conjugated; both from Molecular Probes). After three washes in PBS-T, the cells on coverslips were overlaid onto frosted slides with Fluoromount (Difco). The cells were examined using a Carl Zeiss microscope (Axiovert 200M) attached to a Photometrics Sensys monochrome CCD (charge-coupled-device) camera. Co-localization of the proteins was determined by merging the images using Metamorph software.

### Comparative modelling of mortalin, Bcl-2 and Bcl-xL proteins

The 3D structure of mortalin has not yet been determined experimentally. Though, experimental tertiary structures of Bcl-2 and Bcl-xL has been solved, these structures are still incomplete. We observed that the 3D coordinates of some of the residues, which were experimentally determined to participate in the interaction with Bcl-2 and Bcl-xL, are missing in the reported pdb structures. These coordinates are essential for carrying out detailed computational analysis and hence we generated complete 3D models of these proteins using comparative homology modelling with the help of MODELLER [28]. Bcl-2 was modelled using incomplete structure of Bcl-2 (1GJH) and its most homologous protein structure (Bcl-xL–1LXL, Bcl-W protein–1O0L) as templates while Bcl-xL was modelled using incomplete structure of Bcl-xL (1R2D) and other homologous protein structure (Bcl-xL–1LXL, Bcl-W protein–1O0L) as templates. The structure of human mortalin protein was modelled using Hsp70 (DnaK) chaperone (2KHO) as a template.

### Docking of mortalin protein with Bcl-2 and Bcl-xL structures

Modelled structures of mortalin, Bcl-2 and Bcl-xL were used to perform docking analysis with each other. HADDOCK webserver [29] was used to dock these proteins. It is crucial to provide correct interfacial interacting residual information to HADDOCK in order to get correct docked confirmations of complexes. Results from experimental assays along with information obtained from Interprosurf [30] server were used to identify interacting residues of protein interface. Docked complexes of mortalin-Bcl-2 and mortalin-Bcl-xL were analysed using Protorp server [31] to confirm the interaction sites between the proteins.

### MD (molecular dynamics) simulation of complexes

Stability of interactions between docked complexes of mortalin-Bcl-2 and mortalin-Bcl-xL was verified using 10 ns long MD simulations. Desmond Molecular Dynamics System [32] with OPLS all-atom force field 2005 was used to study the dynamics of protein complexes. Protein–protein complexes were first prepared by protein preparation wizard of Maestro interface (Maestro, version 9.1, Schrödinger, LLC). These prepared structures were then uploaded on to Desmond set up wizard for MD simulations to define the boundary conditions. The systems were then solvated with a triclinic periodic box of TIP4P water molecules. These solvated systems were then neutralized using an appropriate number of counterions. The distances between box wall and protein complexes were set to greater than 10Å to avoid direct interactions with their own periodic image. The complexes were minimized using a maximum of 5000 steps in steepest descent algorithm with a gradient threshold of 25 kcal/mol/Å. The systems were then equilibrated with the default protocol provided in Desmond. Further MD simulations were carried out on the equilibrated systems for 10 ns at constant temperature of 300 K and constant pressure of 101.325 kPa (1 atm≡101.325 kPa) with a time step of 2 fs. During the MD simulations smooth particle-mesh Ewald method was used to calculate long-range electrostatic interactions. 9 Å radius cut-off was used for Coulombic short-range interaction cut-off.

### Analysis of MD simulation

H-bond profiling, interface analysis and conformational analysis of complexes before and after the simulations were used as criterions to analyse the stability of the protein–protein complexes. Desmond Molecular Dynamics System and VMD [33] were used to analyse the MD simulations.

### Calculation of energy contributions of Bcl-2 and Bcl-xL amino acids for probable mortalin binding

Protein sequences in the FASTA format were taken from PubMed. Statistical energy contributions (DD*G*_K_) of individual amino acids were determined within a 13-residue window by applying a motif-based algorithm, a gift from Dr. Bernd Bukau, University of Heidelberg, Germany [34]. DD*G*_K_ value obtained for a defined segment was inversely related to the predicted affinity (the lower the DD*G*_K_ value higher the affinity) between the Hsp70 (mortalin) and the two target proteins, human Bcl-xL (Accn. #CAA80661) and Bcl-2 (Accn. #P10415). Values of adjacent Hsp70-binding segments were averaged and expressed as means±S.D. to determine the regional binding propensities.

## RESULTS

### Bcl-2 and Bcl-xL bind to mortalin: biochemical and bioinformatics evidence

**Figure 1:**
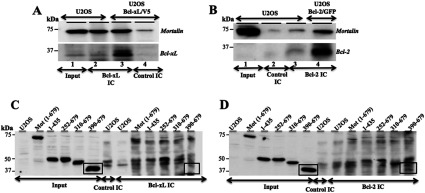
Co-immunoprecipitation of Bcl-2 and Bcl-xL with mortalin Bcl-2 and Bcl-xL IC were probed with anti-mortalin antibody. Control immunoprecipitation was performed with isotype matched irrelevant antibody. (**A**) Mortalin was co-immunoprecipitated with Bcl-xL in control as well as Bcl-xL overexpressing cells (lanes 2 and 3). Control antibody precipitation did not show Bcl-xL, although a faint band of mortalin was observed as a non-specific immunoprecipitation. (**B**) Mortalin was co-immunoprecipitated with Bcl-2 in control as well as Bcl-2 overexpressing cells (lanes 3 and 4). Control antibody precipitation did not show Bcl-2, although a faint mortalin band was observed as a non-specific immunoprecipitation (lane 2). Bcl-2 overexpressing cells showed high amount of mortalin in Bcl-2 complexes (lane 4). (**C** and **D**) Bcl-xL and Bcl-2 were immunoprecipitated from cells transfected with V5-tagged mortalin (full length or deletion mutants as indicated). IC were examined for the presence of mortalin by Western blotting with anti-V5 tag antibody. Deletion mutant containing amino acid residues, 390–679, was not detected in either Bcl-2 (**C**) or Bcl-xL (**D**) IC.

In order to investigate the molecular interactions between Bcl-2 and Bcl-xL with mortalin, we performed co-immunoprecipitation assays. Bcl-2 or Bcl-xL IC from U20S cells were probed with anti-mortalin antibody. As shown in Figures 1 (A) and (B), mortalin was detected in both Bcl-xL and Bcl-2 IC suggesting that these proteins make physical complexes in cells. Lysates from cells transfected with V5-tagged mortalin mutants were next used for immunoprecipitation of Bcl-2 and Bcl-xL. As shown in Figures 1 (C) and (D), we found that the deletion mutant constituting of amino acid residues 1–435, 252–679, 310–679 were able to bind to both Bcl-2 and Bcl-xL. However, the deletion mutant constituting of 390–679 amino acid residues did not bind to either of them suggesting that mortalin 310–390 amino acid residues are involved in its binding to Bcl-2 and Bcl-xL proteins.

**Figure 2:**
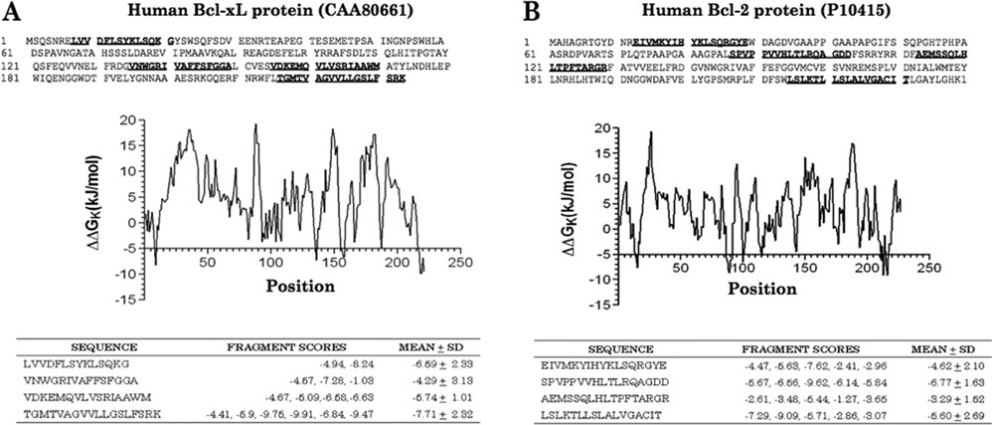
Prediction of binding regions between mortalin and Bcl-xL or Bcl-2 Mortalin binding Bcl-xL (**A**) or Bcl-2 (**B**) regions were predicted by motif-based algorithm [28]. Predicted binding affinities of the fragments are listed in the supplementary tables.

We next undertook a bioinformatics approach to define the interaction between these proteins. Interprosurf server was used to reveal the surface residues of mortalin, Bcl-2 and Bcl-xL protein structures with high probability to interact with other proteins (Supplementary Table S1 available at http://www.bioscirep.org/bsr/033/bsr033e073add.htm). Similarly, mortalin binding regions of Bcl-2 and Bcl-xL were predicted by motif-based algorithm (Figure 2 and Supplementary Table S2 available at http://www.bioscirep.org/bsr/033/bsr033e073add.htm). Residues that could participate in interaction between the proteins were selected on the basis of overlapping residues (Supplementary Tables S1 and S2). Residues were exposed to solvent and found to have high possibility to participate in protein–protein complex formation (Supplementary Table S2). When the modelled structure of mortalin was subjected to *in silico* docking with Bcl-2 and Bcl-xL proteins, based on the experimental data and predicted functional residues, mortalin was able to form complexes with Bcl-2 and Bcl-xL. The analysis of the interface between mortalin-Bcl-2 and mortalin-Bcl-xL complexes revealed the precise details of the residues involved in the interfacial bindings.

**Figure 3:**
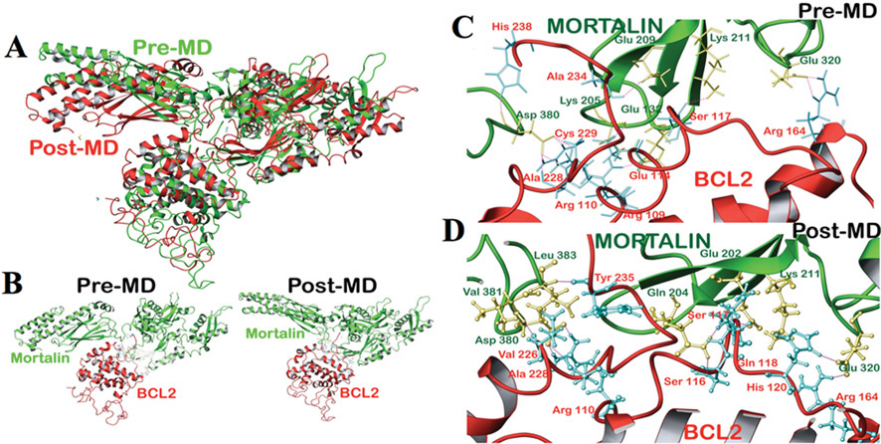
Structural changes in mortalin-Bcl-2 complex after MD simulations (**A**) Superimposition of the structures pre-MD (green) and post-MD (red) simulations. (**B**) Structure of mortalin-Bcl-2 complex pre- and post-MD. Mortalin is represented in green and Bcl-2 is represented in red. H-bond interactions present in mortalin-Bcl-2 complex pre-MD (**C**) and post-MD (**D**). Mortalin is represented in green and Bcl-2 is represented in red.

Residues 373, 376, 377, 380, 381, 382, 283, 284, 386, 387 were identified as residues of interface for mortalin. Similarly, residues 116, 229, 92, 95, 118, 226, 113 and 15, 18, 19, 21, 213, 214, 216, 226, 229, 230 were the residues of interface for Bcl-2 and Bcl-xL proteins, respectively. All these residues occupied more than 50% of solvent accessible area of their respective proteins. Analysis of mortalin-Bcl-2 docked complexes using Protorp server illustrated the presence of 17 hydrogen bonds and 48 salt-bridges at the interface (results not shown). Residues were found to be present at the interface between mortalin and Bcl-2 and involved in the binding interactions (Supplementary Table S3 available at http://www.bioscirep.org/bsr/033/bsr033e073add.htm). These residues matched well with the experimental data. Post-simulation analysis of mortalin-Bcl-2 complex interface revealed that almost all of the residues were conserved at the interface during the MD simulations and found to be involved in binding even after 10 ns (Supplementary Table S4 available at http://www.bioscirep.org/bsr/033/bsr033e073add.htm). Though the conformation of mortalin-Bcl-2 complex was found to be altered after the simulation, the interacting site did not change much and interacting residues were conserved (Figures 3A and 3B). During the simulation, many of the unstable H-bonds were lost and new stable H-bonds appeared (Figures 3C and 3D). We found the occupancy of H-bonds between mortalin and BCL-2 during 10 ns MD simulation (Supplementary Table S5 available at http://www.bioscirep.org/bsr/033/bsr033e073add.htm). It was clear from H-bond occupancy table that most of the bonds were formed between the experimentally reported interfaces. During MD simulation, H-bonds with high occupancy were considered to be stable (shown in bold in Supplementary Table S5). There were 40 salt bridges present at the interface of mortalin-Bcl-2 complex.

**Figure 4:**
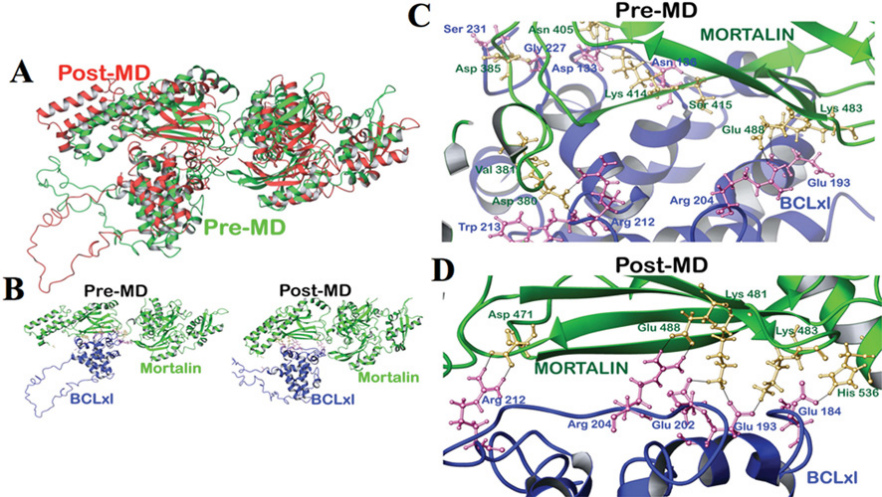
Structural changes in mortalin-Bcl-xL complex after MD simulations **(A)** Superimposition of the structures pre-MD (green) and post-MD (red) simulations. **(B)** Structure of mortalin-Bcl-xL complex pre- and post-MD. Mortalin is represented in green and Bcl-xL is represented in blue. H-bond interactions present in mortalin-Bcl-xL complex: pre-MD **(C)** and post-MD **(D)**. Mortalin is represented in green and Bcl-xL is represented in blue.

Analysis of mortalin-Bcl-xL docked complex by Protorp server revealed the presence of several H-bonds and 31 salt bridges at the interface. Many of the interface residues matched well with the experimental data (Supplementary Table S3). Most of these residues at the interface were also found to be present after 10 ns long MD simulations, suggesting that the residues at the interface are conserved and remained stable during the simulations (Supplementary Table S4). Similar to the mortalin-Bcl-2 complex, mortalin-Bcl-xL complex also changed its conformation during the simulation while keeping the interface unaffected (Figures 4). Few hydrogen bond interactions between mortalin-Bcl-xL post-simulation were found to be different from their structure before the simulation (Figure 4). According to Protorp server analysis, the total number of H-bonds reduced to 12 while the number of salt bridges remained same. There was an occupancy of H-bonds between mortalin and Bcl-2 during 10 ns MD simulation (Supplementary Table S6 available at http://www.bioscirep.org/bsr/033/bsr033e073add.htm; stable H-bonds with high occupancy are highlighted in bold).

### Bcl-2 and Bcl-xL overexpression cause reduction in mortalin leading to an activation of p53 tumour suppressor protein

**Figure 5:**
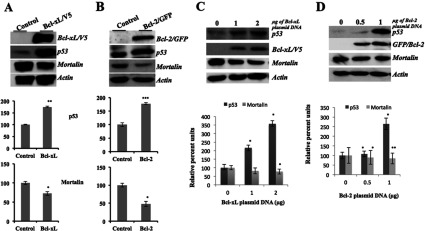
Expression analysis of mortalin and p53 in Bcl-xL and Bcl-2 overexpressing cells Bcl-xL was detected in Western blotting with anti-V5 antibody. Decrease in mortalin (*P<0.05) and increase in p53 (**P<0.005) were observed as a consequence of Bcl-xL overexpression (**A**). Similarly, Bcl-2 (as detected in Western blotting with anti-GFP antibody) overexpressing cells showed decrease in mortalin (*P<0.05) and increase in p53 (***P<0.001) expression (**B**). Dose dependent increase in the level of Bcl-xL (**C**) and Bcl-2 (**D**) caused a serial decrease in mortalin (*P<0.05) and increase in p53 (*P<.05~** *P*<0.005) level. Histograms showing the quantitation of the proteins from three independent experiments (as estimated by ImageJ2x).

In order to elucidate the significance of above described interaction of Bcl-2 and Bcl-xL with mortalin, we overexpressed Bcl-2 and Bcl-xL proteins in U2OS cells. Stably-transfected Bcl-2 and Bcl-xL-overexpressing cells showed decrease in the level of mortalin and increase in p53 (Figures 5A and 5B). Several independent experiments gave consistent results showing statistically significant decrease in mortalin in Bcl-2 and Bcl-xL overexpressing cells. Of note, serial decrease in mortalin was observed in a titration experiment where cells were transiently transfected with increasing amounts of Bcl-2 or Bcl-xL (Figures 5C and 5D). These data revealed that the levels of Bcl-2 and Bcl-xL with mortalin are inversely regulated in cells. Since these proteins have been shown to have anti-apoptotic activity, their inverse relationship might be speculated as an adaptive response in which one apoptotic protein suppresses the other. The detailed mechanism of this inverse regulation such as involvement of transcriptional regulation or proteasomal degradation warrants further studies.

**Figure 6:**
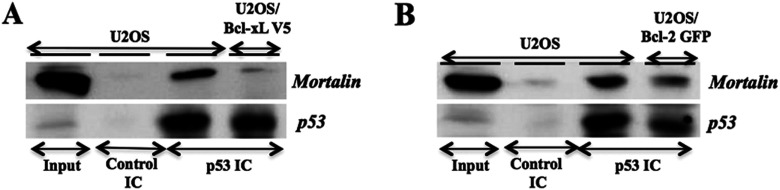
Examination of mortalin-p53 complexes in Bcl-2 and Bcl-xL overexpressing cells Effect of Bcl-xL and Bcl-2 overexpression on mortalin-p53 interactions was determined by examination of mortalin in p53 IC. Stably transfected Bcl-xL (**A**) and Bcl-2-GFP (**B**) cells showed less amount of mortalin in p53 IC.

**Figure 7:**
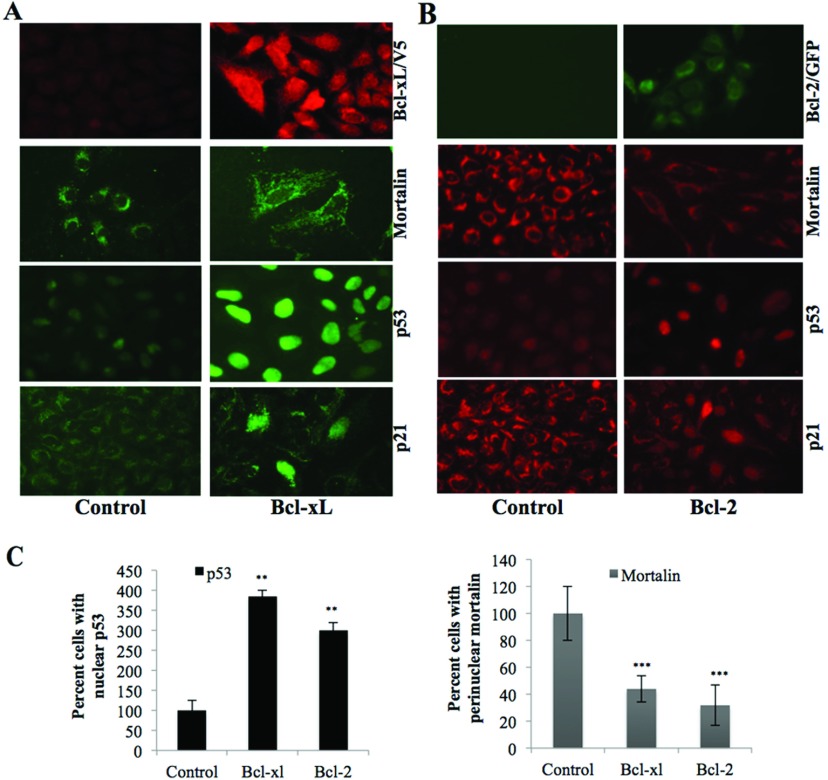
Imaging of mortalin, p53 and p21 in Bcl-xL and Bcl-2 overexpressing cells Immunostaining of Bcl-xL (**A**) or Bcl-2 (**B**) overexpressing cells showing shift in mortalin staining pattern from perinuclear to pancytoplasmic type, enhanced level of expression of nuclear p53 and p21 proteins. (**C**) Quantitation as obtained from three independent experiments is shown.

Mortalin was earlier shown to cause cytoplasmic retention and inactivation of p53 function in human cancer, but not in normal, cells [18,19,35,36]. In light of these data, we anticipated that an increase in p53 protein in Bcl-2 and Bcl-xL overexpressing cells might be due to decreased level of mortalin and abrogation of mortalin-p53 complexes. We examined this hypothesis by immunoprecipitation assay. As shown in Figures 6 (A) and (B), p53 IC were examined for the presence of mortalin by Western blotting using anti-mortalin antibody. We found that the Bcl-2 or Bcl-xL overexpressing cells had less mortalin in p53 IC as compared with the control cells. Furthermore, by immunostaining of cells for Bcl-2, Bcl-xL p53 and mortalin, we indeed detected strong nuclear localization of p53 in Bcl-2 and Bcl-xL overexpressing cells (Figures 7A and 7B). Number of cells with nuclear p53 was quantified. We found that Bcl-2 and Bcl-xL overexpressing cells had nuclear p53 in about 90–95% cells as compared with 25–30% in the control vector-transfected cells suggesting the activation of p53 function that was further confirmed by up-regulation of p53-downstream effector, p21 (Figures 7A and 7B). Taken together with the above data, it was evident that the mechanism of increase in p53 function in Bcl-2/Bcl-xL overexpressing cells involves binding of these proteins to mortalin causing competitive inhibition of mortalin-p53 complex formation resulting in the nuclear translocation and activation of p53 function.

### Bcl-2 and Bcl-xL cause senescence like growth arrest of cells

**Figure 8:**
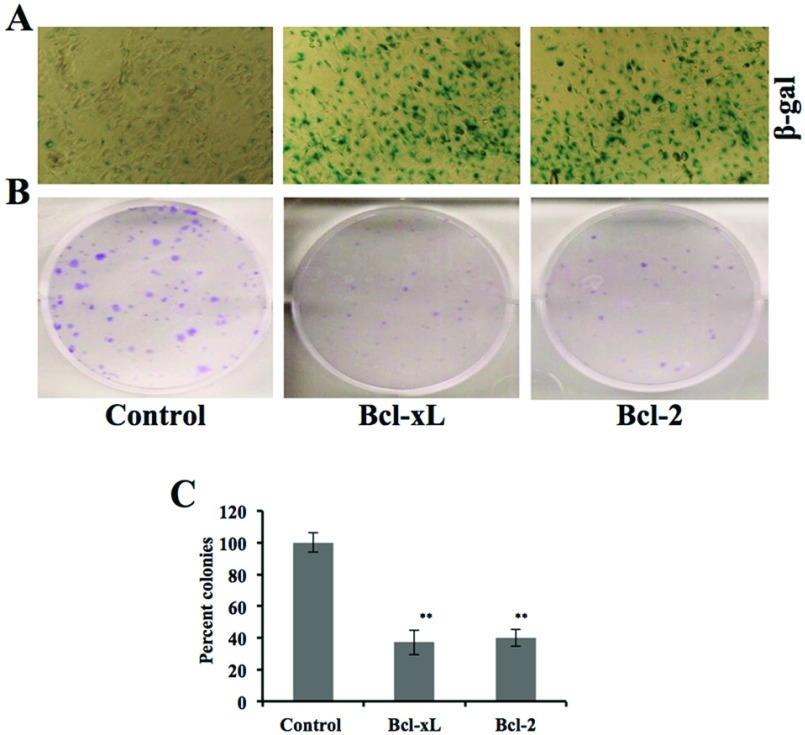
Effects of Bcl-xL and Bcl-2 overexpression on cell proliferation Cells stably transfected with Bcl-xL or Bcl-2 expression plasmids when examined for senescence associated β-gal staining (**A**) showed induction of senescence and decrease in colony forming efficacy (**B**). Quantitation of the colony forming efficiency obtained from three experiments is shown in (**C**) (**P<.005).

Cells overexpressing Bcl-2 and Bcl-xL showed expanded cell morphology and shift in mortalin staining pattern from perinuclear to pancytoplasmic type, characteristic of normal cells. Such shift in mortalin distribution was also reported when cells were induced to senesce by the introduction of single chromosomes, chromosome fragments and low doses of drugs [37–42] suggesting that the Bcl-2/Bcl-xL overexpressing cells might be undergoing senescence as a result of activation of p53 function. Furthermore, in order to examine their senescent status, we performed senescence associated β-gal assay in Bcl-2 and Bcl-xL cells. As shown in Figures 8(A) and 8(B), these cells showed increase in β-gal staining demonstrating induction of senescence. Colony forming efficiency, a reliable indicator of rapid *in vitro* growth and colony forming characteristic of cancer cells, also showed reduction in Bcl-2/Bcl-xL overexpressing cells and was consistent with induction of senescence in these cells. Taken together with the above data on nuclear translocation of p53 and up-regulation of p21, it is suggested that Bcl-2 and Bcl-xL overexpression caused induction of senescence by abrogation of mortalin-p53 interaction and activation of p53 function.

## DISCUSSION

Bcl-2 family proteins are the key players of survival and death pathways in cells. Not only do the different members of this highly conserved protein family possess contrasting functions, the same protein may regulate cell survival and proliferation in opposite directions. For example, depending on their level of expression and interactions, Bcl-xL and Bcl-2 may either inhibit or enhance cell death [43]. Several studies have reported both pro-apoptotic and anti-apoptotic activities of Bcl-2 and Bcl-xL. These are determined by level of expression and its interacting proteins. Interestingly, whereas transient expression of Bcl-xL and Bcl-2 were apoptotic, the stable transfections yielded anti-apoptotic activity. Jung et al. have suggested that the oncogenic potential of Bcl-2 is due to its ability to inhibit senescence as well as apoptosis by inhibiting p53, preventing generation of ROS [27]. Complexity of Bcl-2 and Bcl-xL functionality is reflected by a large number of studies that have been unable to reach consensus on their role in tumorigenesis and tumour-prognosis [44,45]. Knowlton et al. have reported that the overexpression of Bcl-2 in breast cancer cells slowed their *in vitro* growth despite their anti-apoptotic potential that protected cells against doxorubicin, and was predicted to yield favourable outcome in cancer control [46]. Kumazaki et al [47] also reported an induction of premature senescence in Bcl-2 overexpressing normal fibroblasts. These cells were shown to be more sensitive to H_2_O_2_- or doxorubicin-induced cell death, shown to be mediated by activation of MAPK (mitogen-activated protein kinase). Other studies reported delay in cell-cycle progression and DNA replication with overexpression of Bcl-2 [48–50]. The mechanism of induction of growth arrest by either Bcl-2 or Bcl-xL remained unclear in these studies. Our present study to analyse the interaction of these proteins with mortalin demonstrate their direct binding. Furthermore, the interaction involved the amino acid residues in mortalin that were previously shown to be involved to interact with p53 in cancer cells. The interaction between mortalin and Bcl-2/Bcl-xL hence resulted in abrogation of mortalin-p53 binding, nuclear translocation, stabilization and activation of p53 protein causing induction of senescence. The latter was demonstrated by the up-regulation of p21 and senescence-associated β-gal, and decrease in colony forming efficacy in Bcl-2/Bcl-xL overexpressing cells. These results suggest that besides their established role as anti-apoptotic proteins, Bcl-2 and Bcl-xL may reactivate wild-type p53 function resulting in the induction of senescence in cancer cells. Hence a careful consideration is warranted for the use of these proteins and their antagonists in control of cancer cell proliferation and cancer therapeutics.

## Online data

Supplementary data

## References

[1] Petros A. M., Olejniczak E. T., Fesik S. W. (2004). Structural biology of the Bcl-2 family of proteins. Biochim. Biophys. Acta.

[2] Thomadaki H., Scorilas A. (2006). Bcl-2 family of apoptosis-related genes: functions and clinical implications in cancer. Crit. Rev. Clin. Lab. Sci..

[3] Qian J., Voorbach M. J., Huth J. R., Coen M. L., Zhang H., Ng S. C., Comess K. M., Petros A. M., Rosenberg S. H., Warrior U., Burns D. J. (2004). Discovery of novel inhibitors of Bcl-xL using multiple high-throughput screening platforms. Anal. Biochem..

[4] Huang Z. (2000). Bcl-2 family proteins as targets for anticancer drug design. Oncogene.

[5] Deng X., Gao F., Flagg T., May W. S. (2004). Mono- and multisite phosphorylation enhances Bcl-2′s antiapoptotic function and inhibition of cell cycle entry functions. Proc. Natl. Acad. Sci. USA.

[6] Ran Q., Wadhwa R., Kawai R., Kaul S. C., Sifers R. N., Bick R. J., Smith J. R., Pereira-Smith O. M. (2000). Extramitochondrial localization of mortalin/mthsp70/PBP74/GRP75. Biochem. Biophys. Res. Commun..

[7] Liu Y., Liu W., Song X. D., Zuo J. (2005). Effect of GRP75/mthsp70/PBP74/mortalin overexpression on intracellular ATP level, mitochondrial membrane potential and ROS accumulation following glucose deprivation in PC12 cells. Mol. Cell. Biochem..

[8] Kaul S. C., Reddel R. R., Sugihara T., Mitsui Y., Wadhwa R. (2000). Inactivation of p53 and life span extension of human diploid fibroblasts by mot-2. FEBS Lett..

[9] Kaul S. C., Yaguchi T., Taira K., Reddel R. R., Wadhwa R. (2003). Overexpressed mortalin (mot-2)/mthsp70/GRP75 and hTERT cooperate to extend the *in vitro* lifespan of human fibroblasts. Exp. Cell Res..

[10] Xu L., Voloboueva L. A., Ouyang Y., Emery J. F., Giffard R. G. (2009). Overexpression of mitochondrial Hsp70/Hsp75 in rat brain protects mitochondria, reduces oxidative stress, and protects from focal ischemia. J Cereb Blood Flow Metab..

[11] Kaul S. C., Duncan E. L., Englezou A., Takano S., Reddel R. R., Mitsui Y., Wadhwa R. (1998). Malignant transformation of NIH3T3 cells by overexpression of mot-2 protein. Oncogene.

[12] Wadhwa R., Takano S., Kaur K., Deocaris C. C., Pereira-Smith O. M., Reddel R. R., Kaul S. C. (2006). Upregulation of mortalin/mthsp70/Grp75 contributes to human carcinogenesis. Int. J. Cancer.

[13] Yokoyama K., Fukumoto K., Murakami T., Harada S., Hosono R., Wadhwa R., Mitsui Y., Ohkuma S. (2002). Extended longevity of *Caenorhabditis elegans* by knocking in extra copies of hsp70F, a homolog of mot-2 (mortalin)/mthsp70/Grp75. FEBS Lett..

[14] Wadhwa R., Ando H., Kawasaki H., Taira K., Kaul S. C. (2003). Targeting mortalin using conventional and RNA-helicase-coupled hammerhead ribozymes. EMBO Rep..

[15] Wadhwa R., Takano S., Taira K., Kaul S. C. (2004). Reduction in mortalin level by its antisense expression causes senescence-like growth arrest in human immortalized cells. J. Gene Med..

[16] Kimura K., Tanaka N., Nakamura N., Takano S., Ohkuma S. (2007). Knockdown of mitochondrial heat shock protein 70 promotes progeria-like phenotypes in *Caenorhabditis elegans*. J. Biol. Chem..

[17] Wadhwa R., Takano S., Robert M., Yoshida A., Nomura H., Reddel R. R., Mitsui Y., Kaul S. C. (1998). Inactivation of tumor suppressor p53 by mot-2, a hsp70 family member. J. Biol. Chem..

[18] Kaul S. C., Aida S., Yaguchi T., Kaur K., Wadhwa R. (2005). Activation of wild type p53 function by its mortalin-binding, cytoplasmically localizing carboxyl terminus peptides. J. Biol. Chem..

[19] Ma Z., Izumi H., Kanai M., Kabuyama Y., Ahn N. G., Fukasawa K. (2006). Mortalin controls centrosome duplication via modulating centrosomal localization of p53. Oncogene.

[20] Lu W. J., Lee N. P., Kaul S. C., Lan F., Poon R. T., Wadhwa R., Luk J. M. (2011). Mortalin-p53 interaction in cancer cells is stress dependent and constitutes a selective target for cancer therapy. Cell Death Differ..

[21] Lu W. J., Lee N. P., Kaul S. C., Lan F., Poon R. T. P., Wadhwa R., Luk J. M. (2011). Induction of mutant p53-dependent apoptosis in human hepatocellular carcinoma by targeting stress protein mortalin. Int. J. Cancer.

[22] Bruschi S. A., Lindsay J. G. (1994). Mitochondrial stress protein actions during chemically induced renal proximal tubule cell death. Biochem. Cell Biol..

[23] Qu M., Zhou Z., Xu S., Chen C., Yu Z., Wang D. (2011). Mortalin overexpression attenuates beta-amyloid-induced neurotoxicity in SH-SY5Y cells. Brain Res..

[24] Mihara M., Moll U. M. (2003). Detection of mitochondrial localization of p53. Methods Mol. Biol..

[25] Deng X., Gao F., Flagg T., Anderson J., May W. S. (2006). Bcl2′s flexible loop domain regulates p53 binding and survival. Mol. Cell Biol..

[26] Petros A. M., Gunasekera A., Xu N., Olejniczak E. T., Fesik S. W. (2004). Defining the p53 DNA-binding domain/Bcl-xL-binding interface using NMR. FEBS Lett..

[27] Jung M. S., Jin D. H., Chae H. D., Kang S., Kim S. C., Bang Y. J., Choi T. S., Choi K. S., Shin D. Y. (2004). Bcl-xL and E1B-19K proteins inhibit p53-induced irreversible growth arrest and senescence by preventing reactive oxygen species-dependent p38 activation. J. Biol. Chem..

[28] Fiser A., Sali A. (2003). Modeller: generation and refinement of homology-based protein structure models. Methods Enzymol..

[29] Dominguez C., Boelens R., Bonvin A. M. (2003). HADDOCK: a protein-protein docking approach based on biochemical or biophysical information. J. Am. Chem. Soc..

[30] Negi S. S., Schein C. H., Oezguen N., Power T. D., Braun W. (2007). InterProSurf: a web server for predicting interacting sites on protein surfaces. Bioinformatics.

[31] Reynolds C., Damerell D., Jones S. (2009). ProtorP: a protein-protein interaction analysis server. Bioinformatics.

[32] Desmond Molecular Dynamics System (2010). Version 2.4, D.E. Shaw Research, New York, NY, 2010. Maestro-Desmond Interoperability Tools, Version 2.4.

[33] Humphrey W., Dalke A., Schulten K. (1996). VMD–visual molecular dynamics’. J. Mol. Graphics.

[34] Rudiger S., Germeroth L., Schneider-Mergener J., Bukau B. (1997). Substrate specificity of the DnaK chaperone determined by screening cellulose-bound peptide libraries. EMBO J..

[35] Walker C., Bottger S., Low B. (2006). Mortalin-based cytoplasmic sequestration of p53 in a nonmammalian cancer model. Am. J. Pathol..

[36] Wadhwa R., Yaguchi T., Hasan M. K., Mitsui Y., Reddel R. R., Kaul S. C. (2002). Hsp70 family member, mot-2/mthsp70/GRP75, binds to the cytoplasmic sequestration domain of the p53 protein. Exp. Cell Res..

[37] Bertram M. J., Berube N. G., Hang-Swanson X., Ran Q., Leung J. K., Bryce S., Spurgers K., Bick R. J., Baldini A., Ning Y. (1999). Identification of a gene that reverses the immortal phenotype of a subset of cells and is a member of a novel family of transcription factor-like genes. Mol. Cell Biol..

[38] Nakabayashi K., Ogata T., Fujii M., Tahara H., Ide T., Wadhwa R., Kaul S. C., Mitsui Y., Ayusawa D. (1997). Decrease in amplified telomeric sequences and induction of senescence markers by introduction of human chromosome 7 or its segments in SUSM-1. Exp. Cell Res..

[39] Wadhwa R., Sugihara T., Yoshida A., Nomura H., Reddel R. R., Simpson R., Maruta H., Kaul S. C. (2000). Selective toxicity of MKT-077 to cancer cells is mediated by its binding to the hsp70 family protein mot-2 and reactivation of p53 function. Cancer Res..

[40] Widodo N., Deocaris C. C., Kaur K., Hasan K., Yaguchi T., Yamasaki K., Sugihara T., Ishii T., Wadhwa R., Kaul S. C. (2007). Stress chaperones, mortalin, and pex19p mediate 5-aza-2′ deoxycytidine-induced senescence of cancer cells by DNA methylation-independent pathway. J. Gerontol. A Biol. Sci. Med. Sci..

[41] Widodo N., Kaur K., Shrestha B. G., Takagi Y., Ishii T., Wadhwa R., Kaul S. C. (2007). Selective killing of cancer cells by leaf extract of Ashwagandha: identification of a tumor-inhibitory factor and the first molecular insights to its effect. Clin. Cancer Res.

[42] Grover A., Priyandoko D., Gao R., Shandilya A., Widodo N., Bisaria V. S., Kaul S. C., Wadhwa R., Sundar D. (2012). Withanone binds to mortalin and abrogates mortalin-p53 complex: computational and experimental evidence. Int. J. Biochem. Cell Biol..

[43] Kim R. (2005). Unknotting the roles of Bcl-2 and Bcl-xL in cell death. Biochem. Biophys. Res. Commun..

[44] Shinoura N., Yoshida Y., Nishimura M., Muramatsu Y., Asai A., Kirino T., Hamada H. (1999). Expression level of Bcl-2 determines anti- or proapoptotic function. Cancer Res..

[45] Watanabe J., Kushihata F., Honda K., Sugita A., Tateishi N., Mominoki K., Matsuda S., Kobayashi N. (2004). Prognostic significance of Bcl-xL in human hepatocellular carcinoma. Surgery.

[46] Knowlton K., Mancini M., Creason S., Morales C., Hockenbery D., Anderson B. O. (1998). Bcl-2 slows *in vitro* breast cancer growth despite its antiapoptotic effect. J. Surg. Res..

[47] Kumazaki T., Sasaki M., Nishiyama M., Teranishi Y., Sumida H., Eboshima A., Mitsui Y. (2003). Life span shortening of normal fibroblasts by overexpression of BCL-2: a result of potent increase in cell death. Exp. Cell Res..

[48] Borner C. (1996). Diminished cell proliferation associated with the death protective activity of Bcl-2. J. Biol. Chem..

[49] Mazel S., Burtrum D., Petrie H. T. (1996). Regulation of cell division cycle progression by Bcl-2 expression: a potential mechanism for inhibition of programmed cell death. J. Exp. Med..

[50] Vairo G., Innes K. M., Adams J. M. (1996). Bcl-2 has a cell cycle inhibitory function separable from its enhancement of cell survival. Oncogene.

