# Supplementary material for: Molecular interactions of Bcl-2 and Bcl-xL with mortalin: identification and functional characterization
Source: Biosci Rep. 2013 Oct 16;33(5):e00073. doi: 10.1042/BSR20130034 (PMC3797589; doi:10.1042/BSR20130034)
Supplement: Supplementary data [file bsr033e073add.pdf]

Biosci. Rep. (2013) / **33** / art:e00073 / doi 10.1042/BSR20130034

OPEN ACCESS

Original Paper

SUPPLEMENTARY DATA

Molecular interactions of Bcl-2 and Bcl-xL with mortalin: identification and functional characterization

Nishant SAXENA*[1], Shashank P. KATIYAR†[1], Ye LIU*, Abhinav GROVER†, Ran GAO*, Durai SUNDAR†[2], Sunil C. KAUL*[2] and Renu WADHWA*[2]

*National Institute of Advanced Industrial Science and Technology (AIST), Central 4, 1-1-1 Higashi, Tsukuba Science City 305-8562, Japan, and †Department of Biochemical Engineering and Biotechnology, Indian Institute of Technology (IIT) Delhi, Hauz Khas, New Delhi 110016, India

Table S1 Predicted amino acid residues of proteins that participate in docking

Mortalin	Bcl-2	Bcl-xL
320–400	12–29, 87-103, 113–119, 215–last	8–21, 135–149, 145–170, 216–last

Table S2 List of functional residues at the interface of mortalin, Bcl-2 and Bcl-xL proteins

Protein	Residues
Mortalin	Val-373,Gly-376, Asp-377, Asp-380, Val-381, Leu-382, Leu-383, Leu-384, Val-386, Thr-387
Bcl-2	Ser-116, Cys-229, Val-92, Leu-95, Gln-118, Val-226, Ala-113
Bcl-xL	Tyr-15, Ser-18, Gln-19, Gly-21, Trp-213, Phe-214, Thr-216, Leu-226, Leu-229, Phe-230

Table S3 Interfacial residues in docked protein–protein complexes before MD simulations

Mortalin–Bcl-2 Complex		Mortalin–Bcl-xL Complex	
**Mortalin**	**Bcl-2**	**Mortalin**	**Bcl-xL**
Asp-145, Arg-148, Glu-182, Asp-318, Glu-320, Pro-411, Thr-412, Lys-413, Lys-481, Lys-483, Gly-486, Glu-488	Arg-109, Arg-110, Ala-113, Glu-114, Ser-117, Gln-118, His-120, Arg-164, Pro-208, Phe-212, Leu-215, Ser-216, Thr-219, Leu-220, Ala-224, Leu-225, Val-226, Gly-227, Ala-228, Cys-229, Gly-233, Ala-234, Tyr-235, Leu-236, His-238, Lys-239	Thr-379,Asp-380, Val-381, Leu-382, Leu-383, Leu-384, Asp-385, Gln-463, Glu-465, Asp-469, Asp-471, Ala-472, Asn-473	Trp-181, Asn-185, Gly-186, Asp-189, Thr-190, Glu-193, Leu-194, Tyr-195

[1] These authors contributed equally to this work.
[2] Correspondence may be addressed to either of these authors (email renu-wadhwa@aist.go.jp or s-kaul@aist.go.jp or sundar@dbeb.iitd.ac.in).

© 2013 The Author(s) This is an Open Access article distributed under the terms of the Creative Commons Attribution Licence (CC-BY) (http://creativecommons.org/licenses/by/3.0/) which permits unrestricted use, distribution and reproduction in any medium, provided the original work is properly cited.

Bioscience Reports www.bioscirep.org

Table S4 Interfacial residues in docked protein–protein complexes after MD simulations

Mortalin-Bcl-2 complex		Mortalin-Bcl-xL complex	
**Mortalin**	**Bcl-2**	**Mortalin**	**Bcl-xL**
Arg-148, Lys-152, Glu-202, Gln-204, Lys-205, Gly-206, Val-207, Phe-208, Glu-209, Val-210, Lys-211, Val-378, Thr-379, Asp-380, Val-381, Leu-382, Leu-383, Leu-384, Asp-385	Arg-110, Glu-114, Ser-117, Gln-118, His-120, Arg-164, Glu-165, Leu-223, Val-226, Gly-237, His-238, His-239	Gln-204, Lys-205, Gly-206, Val-207, Phe-208, Glu-209, Leu-403, Ile-404, Asn-405, Arg-406, Asn-407, Thr-408, Pro-411, Thr-412, Lys-413, Lys-414, Ser-415, Gln-416, Val-417, Phe-418, Asp-471, Ala-472, Asn-473	Trp-181, Glu-184, Asn-185, Gly-186, Asp-189, Thr-190, Glu-193, Leu-194, Tyr-195

Table S5 H-bond occupancy of mortalin–Bcl-2 complex during MD simulation Stable H-bonds with high occupancy are shown in bold letters.

Mortalin	Bcl-2	Occupancy
**Gln-204-side**	**Ser-117-side**	**42.28%**
Val-381-main	Val-226-main	1.15%
**Asp-380-side**	**Arg-110-side**	**100.00%**
**Glu-202-side**	**Ser-117**-side	**69.08%**
**Glu-320-side**	**Arg-164-side**	**37.97%**
Val-210-main	Gln-118-side	11.60%
**Asp-385-side**	**Lys-239-side**	**34.47%**
Lys-205-main	Glu-114-side	6.62%
**Gly-206-main**	**Glu-114-side**	**25.60%**
Glu-182-side	Arg-109-side	1.25%
Lys-413-side	Leu-215-main	5.94%
Glu-209-side	Tyr-235-main	0.72%
Asp-380-side	Ala-228-main	2.83%
Glu-320-side	His-120-side	1.34%
Glu-209-side	Gln-118-side	8.15%
**Glu-209-side**	**Ala-234-main**	**32.89%**
Asp-380-main	Hie-238-side	0.10%
Val-207-main	Glu-114-side	7.19%
Lys-413-side	Thr-219-side	0.05%
Asp-380-side	Cys-229-side	0.05%
Thr-379-side	Arg-110-side	0.38%
Lys-481-side	Asp-211-side	1.01%
Thr-379-side	Arg-110-side	0.14%
Val-210-main	Tyr-235-side	2.25%

© 2013 The Author(s) This is an Open Access article distributed under the terms of the Creative Commons Attribution Licence (CC-BY) (http://creativecommons.org/licenses/by/3.0/) which permits unrestricted use, distribution and reproduction in any medium, provided the original work is properly cited.

Table S6 H-bond occupancy of mortalin–Bcl-xL complex during MD simulation Stable H-bonds with high occupancy are shown in bold letters.

Mortalin	Bcl-xL	Occupancy
**Lys-481-side**	**Glu-202-side**	**34.52%**
Lys-483-side	Glu-193-side	0.96%
**Glu-488-side**	**Arg-204-side**	**100.00%**
**Asp-471-side**	**Arg-212-side**	**100.00%**
Lys-483-side	Glu-193-main	0.62%
Asn-405-side	Asp-133-side	5.32%
Asp-380-side	Arg-212-side	0.62%
Asp-380-side	Trp-213-main	8.44%
Ser-415-main	Asn-136-side	3.79%
Asp-385-side	Ser-231-side	2.73%
Gln-463-side	Thr-190-side	1.25%
Lys-414-side	Asn-136-side	0.14%
Val-417-main	Asn-185-side	8.34%
**Asp-385-side**	**Gly-227-main**	**20.13%**
**Lys-414-side**	**Asp-133-side**	**25.60%**
Gln-463-side	Thr-190-side	5.80%
Asn-405-side	Lys-233-side	0.24%
Asn-473-side	Arg-212-side	2.73%
Asp-385-side	Ser-231-main	4.46%
Asn-407-side	Lys-233-side	1.34%
Arg-406-main	Lys-233-side	6.57%
Gln-463-side	Asn-185-main	1.73%
Gln-416-side	Trp-181-side	0.14%
Arg-209-side	Asp-179-main	0.05%
Gln-463-side	Asp-189-side	0.91%
Asn-407-side	Ser-231-side	0.05%
Lys-483-side	Thr-190-side	3.88%
**Arg-209-side**	**Glu-182-side**	**67.02%**
Lys-481-side	Glu-193-side	18.98%
Lys-483-side	Thr-190-main	0.14%
Gln-416-side	Glu-184-side	0.10%
Arg-209-side	Lys-180-main	0.43%
Gln-463-side	Glu-184-main	0.10%
Gln-463-side	Glu-184-side	0.05%
Glu-465-side	Asn-185-side	0.14%
Lys-483-side	Asn-185-main	0.91%
Lys-413-side	Thr-219-side	0.34%
**His-536-side**	**Glu-184-side**	**33.27%**
Glu-543-side	Gln-183-side	0.24%
Lys-413-side	Phe-214-main	0.14%

Received 25 March 2013/28 May 2013; accepted 3 June 2013

Published as Immediate Publication 20 September 2013, doi 10.1042/BSR20130034

© 2013 The Author(s) This is an Open Access article distributed under the terms of the Creative Commons Attribution Licence (CC-BY) (http://creativecommons.org/licenses/by/3.0/) which permits unrestricted use, distribution and reproduction in any medium, provided the original work is properly cited.
